# Ca^2+^ activated Cl^−^ channels as targets for analgesics

**DOI:** 10.18632/oncotarget.18354

**Published:** 2017-06-02

**Authors:** Isabella Salzer, Klaus Schicker, Stefan Boehm

**Affiliations:** Centre for Physiology and Pharmacology, Medical University of Vienna, Waehringerstrasse, Vienna, Austria

**Keywords:** TRPV_1_ channels, Ca^2+^-activated Cl^−^ channels, anoctamins, sensory neurons, pain

The protein *Discovered On Gastrointestinal stromal tumors* (GIST) *protein 1* (DOG1) is a biomarker used in the diagnosis of this type of neoplasm. Nevertheless, high DOG1 expression levels cannot only be detected in GIST, but also in a considerable number of other tumors [1]. DOG1 is also known as *ANOctamin 1* (ANO1) and *TransMEMbrane protein with unknown function 16A* (TMEM16A) and belongs to a superfamily of proteins that operate either as Ca^2+^-activated Cl^−^ channels (CaCCs) or as phospholipid scramblases. CaCCs formed by these proteins may subserve a variety of biological functions that include, for instance, blood pressure regulation, lung ventilation, saliva production and intestinal Cl^−^ secretion [1]. Anoctamins are also involved in tumorigenesis and tumor proliferation, but whether these actions are related to a channel mode of operation remains controversial. Nevertheless, in several instances ANO1 channel blocking agents were found to reduce tumor progression [1].

**Figure 1:**
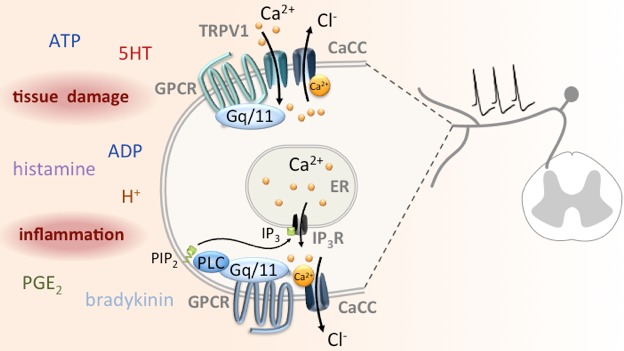
Mechanisms of CACC activation in nociceptors Due to mechanical damage or inflammation, constituents of the inflammatory soup are released and activate GPCRs on nociceptors. Via heterotrimeric Gq/11 type G proteins and phospholipase C (PLC), intracellular Ca^2+^ levels are raised through the gating of either inositol trisphosphate (IP_3_) receptors in the endoplasmic reticulum or TRPV1 channels in the plasma membrane. This Ca^2+^ elevation leads to the opening of ANO1 or possibly other CaCCs, and the resulting Cl^−^ efflux causes depolarization and increased action potential propagation towards the spinal cord.

A recent addition to this list of anoctamin functions is pain perception. First, ANO1 was revealed to contribute to the mechanisms that underlie the algogenic action of bradykinin: the peptide was reported to activate depolarizing currents through ANO1 in nociceptive neurons by releasing Ca^2+^ from the endoplasmic reticulum (Figure 1) [2]. Bradykinin and numerous other signaling molecules, such as protons, prostanoids, nucleotides, histamine, and serotonin, are released from cells surrounding the nociceptors under conditions of tissue damage and inflammation. Together, these mediators are termed “inflammatory soup” and as such they are known to enhance the excitability of sensory neurons. The combined action of an experimental inflammatory soup containing bradykinin, prostaglandin E2, histamine, and serotonin on the excitability of nociceptive neurons was largely attenuated or even abolished when these neurons did not express ANO1 [3]. Most recently, serotonin on its own has been shown to excite nociceptive sensory neurons through an activation of CaCCs [4]. This action was mediated by 5HT2C receptors and did also involve an activation of TRPV1 channels. This confirmed previous reports demonstrating that TRPV1 and ANO1 channels can directly interact with each other [5]. Furthermore, blockers of ANO1 channels were shown to reduce the excitatory actions of the TRPV1 channel activator capsaicin on sensory neurons, on the one hand, and the nocifensive behavior of mice in response to TRPV1 activation, on the other hand [5, 6]. The presumed underlying process is the gating of CaCCs by Ca^2+^ which enters the neurons via TRPV1 channels (Figure 1). Since sensory neurons accumulate high intracellular Cl^−^ concentrations, the opening of a Cl^−^ channel leads to Cl^−^ efflux, thereby triggering membrane depolarization and a resultant increase in excitability [7].

A large number of inflammatory mediators including bradykinin, nucleotides (ATP and ADP), prostaglandin E2 (PGE_2_) and serotonin (5HT) facilitate the gating of TRPV1 channels through an activation of appropriate G protein coupled receptors (GPCRs; Figure 1). In the case of serotonin, activation of 5HT2A, 5HT2B, and 5HT2C receptors can enhance currents through TRPV1 channels, but only via the latter receptor serotonin did cause gating of CaCCs and increased the excitability of sensory neurons [4]. Obviously, certain, but not all, GPCRs are posed in a strategic position to simultaneously control TRPV1 channels and CaCCs which then mediate inflammatory pain in collaboration (Figure 1).

ANO1 has also been found to play a role in thermal pain [3, 7]. Similarly to TRPV1 and other TRP channels, ANO1 can be activated by heat, and knock-out of ANO1 as well as appropriate blockers do reduce behavioral responses to thermal stimulation [7]. Whether the sensation of painful heat may also rely on an interaction between CaCCs and TRP channels has not been elucidated. Nevertheless, analgesic actions of agents that block CaCCs in a rather unspecific manner have been documented in different animal models of inflammatory and thermal pain [2, 5, 6, 7]. Most recently, pharmacological characteristics of the first selective small molecule blockers of ANO1 have been reported [8]. Hence, we are on our road towards subtype selective CaCC inhibitors as novel analgesics that might also prove beneficial with respect to anticancer activity.
